# The role of the serotonin transporter in prefrontal cortex glutamatergic signaling following short‐ and long‐access cocaine self‐administration

**DOI:** 10.1111/adb.12896

**Published:** 2020-03-18

**Authors:** Lucia Caffino, Francesca Mottarlini, Boyd Van Reijmersdal, Francesca Telese, Michel M.M. Verheij, Fabio Fumagalli, Judith R. Homberg

**Affiliations:** ^1^ Department of Pharmacological and Biomolecular Sciences Università degli Studi di Milano Milan Italy; ^2^ Department of Cognitive Neuroscience, Division of Molecular Neurogenetics, Donders Institute for Brain, Cognition and Behaviour Radboud University Nijmegen Medical Centre Nijmegen The Netherlands

**Keywords:** glutamate, infralimbic cortex, prelimbic cortex, SERT

## Abstract

Vulnerability to drug addiction relies on substantial individual differences. We previously demonstrated that serotonin transporter knockout (SERT^−/−^) rats show increased cocaine intake and develop signs of compulsivity. However, the underlying neural mechanisms are not fully understood. Given the pivotal role of glutamate and prefrontal cortex in cocaine‐seeking behavior, we sought to investigate the expression of proteins implicated in glutamate neurotransmission in the prefrontal cortex of naïve and cocaine‐exposed rats lacking SERT. We focused on the infralimbic (ILc) and prelimbic (PLc) cortices, which are theorized to exert opposing effects on the control over subcortical brain areas. SERT^−/−^ rats, which compared to wild‐type (SERT^+/+^) rats show increased ShA and LgA intake short‐access (ShA) and long‐access (LgA) cocaine intake, were sacrificed 24 h into withdrawal for ex vivo molecular analyses. In the ILc homogenate of SERT^−/−^ rats, we observed a sharp increase in glial glutamate transporter 1 (GLT‐1) after ShA, but not LgA, cocaine intake. This was paralleled by ShA‐induced increases in GluN1, GluN2A, and GluN2B NMDA receptor subunits and their scaffolding protein SAP102 in the ILc homogenate, but not postsynaptic density, of these knockout animals. In the PLc, we found no major changes in the homogenate; conversely, the expression of GluN1 and GluN2A NMDA receptor subunits was increased in the postsynaptic density under ShA conditions and reduced under LgA conditions. These results point to SERT as a critical regulator of glutamate homeostasis in a way that differs between the subregions investigated, the duration of cocaine exposure as well as the cellular compartment analyzed.

## Graphical abstract

Serotonin transporter deletion is associated with increase cocaine intake. We sought to investigate the underlying mechanisms at the level of the glutamate synapse in the prelimbic and infralimbic cortices. We observed that serotonin transporter deletion is associated with dysregulation of the serotonergic synapse in a region specific manner.

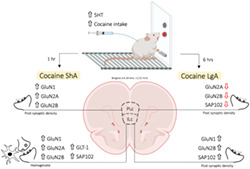

## 1 INTRODUCTION

Drug addiction is classified as a compulsive and relapsing psychiatric disease, characterized by the transition from limited to compulsive use. Such transition may result from a negative emotional state that becomes manifest during abstinence.^1^ One molecule that plays a key role in this transition to compulsivity is serotonin.^2^ The plasmalemmal serotonin transporter (SERT), whose main function is to uptake serotonin back into the presynaptic terminal, plays a critical role in negative emotionality as well as drug intake. In fact, the lack of SERT in animals has been closely associated with increased anxiety^3^ and with a higher intake of cocaine.^4^ We have recently focused our attention on cocaine self‐administration behaviors using different conditions of psychostimulant intake that mimic both limited or compulsive drug use, the so‐called short‐access (ShA) or long‐access (LgA) cocaine, respectively.^2^, ^4^, ^5^, ^6^ We found that cocaine intake was increased in SERT knockout (SERT^−/−^) rats under both ShA and LgA conditions and that anxiety was increased 24 h into withdrawal from both conditions. This implies that increases in both regular and compulsive cocaine intake is, at least in part, driven by increased anxiety, adding critical information with respect to the neural mechanisms involved in the pathophysiology underlying addictive behavior.

The medial prefrontal cortex (mPFC) plays an essential role in drug seeking, abstinence, and relapse, as inferred from rodent studies.^7^, ^8^, ^9^ In addition, it is well established that the mPFC plays also a critical role in decision‐making and behavioral flexibility. mPFC receives excitatory glutamatergic inputs from the sensory systems and adjusts them to the signals received from the hippocampus and amygdala.^10^ When the activity of mPFC is dysregulated, the balance between the promotion or inhibition of a given function may be altered as well.^11^ The glutamate system plays a key role in the functioning of the mPFC. For instance, withdrawal from cocaine self‐administration is associated with an increase in extracellular glutamate levels^12^ and an upregulation of the GluN2B receptor subunit.^13^ Furthermore, we have previously shown that repeated cocaine injections render the glutamatergic synapse of the mPFC sensitive to stress, an event that is suggested to play a central role in the reinstatement of cocaine seeking.^14^, ^15^ To this end, investigating the role of mPFC to drug seeking is critical as, for instance, cortical hypofrontality appears to be associated with cognitive impairments in cocaine addicts contributing to compulsive drug seeking.^16^, ^17^, ^18^, ^19^

From an anatomical point of view, the mPFC is divided into infralimbic (ILc) and prelimbic (PLc) cortices. Notably, although these two brain regions are in close proximity within the brain, they play different roles, sometimes even opposite, highlighting a functional dichotomy of these two brain regions. For instance, the PLc is recruited for the initiation of a conditioned fear response, whereas the ILc initiates, through extinction training, the suppression of conditioned fear responding.^20^, ^21^ Similar to fear conditioning, these two brain regions serve distinct functions in the response to the psychostimulant cocaine. In fact, the PLc appears to be critical for the initiation of cocaine seeking, whereas the ILc is engaged by extinction learning to suppress cocaine seeking.^22^, ^23^, ^24^ Surprisingly, we know very little about cocaine‐induced plasticity in the ILc and PLc, especially in terms of changes of excitatory signaling.

We have previously investigated glutamate homeostasis in the habenula of SERT^−/−^ rats.^25^ It has been established that cocaine enhances the glutamatergic transmission in those neurons projecting from habenula to the rostral tegmental area^26^ that, in turn, influence the serotonergic tone via inhibition of the dorsal raphe nucleus.^27^ We found that the deletion of SERT altered cocaine‐induced glutamate homeostasis of the habenula. However, the habenula does not function in isolation, and evidence exists showing that glutamatergic determinants of the mPFC are altered in SERT^−/−^ rats, both at baseline and following cocaine exposure.^5^, ^28^ To the best of our knowledge, no evidence exists on the role of SERT in the regulation of the glutamatergic synapse in the IL versus PL cortical subregions.

To fill this gap, SERT^+/+^ and SERT^−/−^ rats were exposed to both ShA (1 h/day, 0.5 mg/kg/infusion) or LgA cocaine self‐administration protocol (6 h/day, 0.5 mg/kg/infusion) to mimic limited consumption or the loss of control over drug intake, respectively.^29^, ^30^ By comparing cocaine‐naive rats to rats exposed to ShA or LgA of cocaine, we evaluated the effects of SERT removal on critical determinants of glutamate homeostasis such as (1) the glial glutamate transporter 1 (GLT‐1), which is responsible of the termination of glutamate neurotransmission by mediating reuptake of glutamate back into the presynaptic terminal and (2) the different subunits of the NMDA receptor (GluN1, GluN2A, and GluN2B), which represent the main glutamate receptor complex responsible of calcium influx into the cell,^31^ as well as the scaffolding proteins PSD95 and SAP102, which are responsible of the anchoring of NMDA receptor tight to the postsynaptic density.^32^ We evaluated the expression of these molecules in the whole homogenate, which informs us primarily about translational changes, as well as in the postsynaptic density (PSD), which gives us a clue of synaptic localization of receptors and the respective scaffolding proteins. In doing so, we took advantage of unused brain material collected from naïve and ShA and LgA cocaine SERT^−/−^ as well as wild‐type rats in a previous study^25^ and examined the glutamate synapse in the ILc and PLc.

## 2 MATERIAL AND METHODS

### 2.1 Animals

SERT^−/−^ rats (SLC6A41Hubr) were generated by N‐ethyl‐N‐nitrosurea (ENU) induced mutagenesis^33^ and outcrossed with commercially available Wistar rats (Harlan, Ter Horst, the Netherlands) for at least 10 generations.^34^ Male SERT^−/−^ rats and their wild‐type (SERT^+/+^) counterparts were subjected to short‐ and long‐access (ShA and LgA) cocaine self‐administration according to the procedures derived from Caffino et al. (ShA: SERT^+/+^: *n* = 6, SERT^−/−^: *n* = 6 and LgA: SERT^−/−^: *n* = 6, SERT^−/−^: *n* = 6).^35^ All procedures were carried out in agreement with the current National Research Council Guide for the Care and Use of Laboratory Animals and were approved by local Institutional Animal Care and Use Committees. All efforts were made to reduce the number of animals used and their suffering.

### 2.2 Cocaine self‐administration

Briefly, 1 week after surgery, rats were trained to self‐administer cocaine (0.5 mg/kg/infusion) under a fixed ratio 1 (FR1) schedule of reinforcement for details^6^, ^9^ Two days after cocaine self‐administration training, rats were allowed to self‐administer cocaine during daily 6‐h sessions (extended or LgA group of rats) or 1‐h sessions (limited of ShA group of rats) for a total of 15 days.^30^ Additional groups of cocaine‐naive SERT^−/−^ and SERT^+/+^ rats also underwent intravenous catheterization, were handled daily, and received daily infusion of heparinized saline but were not exposed to the self‐administration chambers.^6^, ^9^

### 2.3 Tissue collection

**FIGURE 1:**
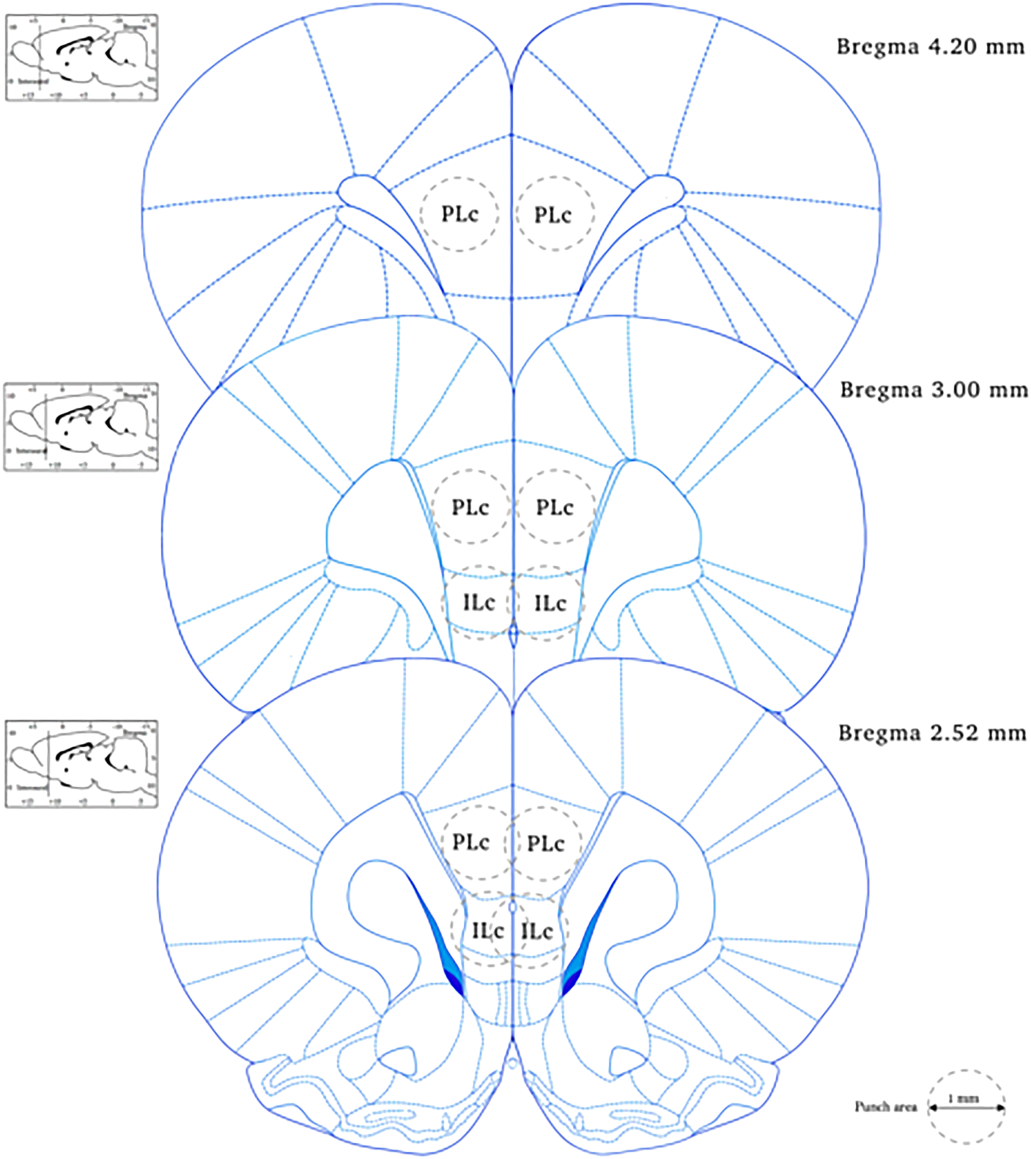
Specific coordinates of infralimbic (ILc) and prelimbic (PLc) cortices

Twenty‐four hours following the last cocaine self‐administration session, rats were sacrificed by decapitation, brains were quickly collected and stored at −80°C. Using the rat brain atlas,^36^ the PLc and ILc (coordinates between bregma +4.20 mm and bregma +2.52 mm, Figure 1) were punched from frozen brain sections of 220 μm using a sterile 1‐mm‐diameter needle.^37^ Punches from the right and left hemisphere were pooled. Prelimbic and infralimbic tissue was stored at −80°C until being processed for molecular analysis (see below).

### 2.4 Protein extraction and western blot analyses

Proteins were extracted as previously described^38^ with minor modifications. Briefly, bilateral punches of ILc and PLc regions of mPFC were homogenized in a teflon‐glass potter in cold 0.32‐M sucrose buffer pH 7.4 containing 1‐mM HEPES, 1‐mM MgCl2, 1‐mM NaHCO3, and 0.1‐mM PMSF, in presence of commercial cocktails of protease (Roche, Monza, Italy) and phosphatase (Sigma‐Aldrich, Milan, Italy) inhibitors and an aliquot of each homogenate was then sonicated. The remaining homogenate was centrifuged at 13,000 g for 15 min obtaining a pellet. This pellet was resuspended in buffer containing 75‐mM KCl and 1% Triton X‐100 and centrifuged at 100,000 × g for 1 h. The resulting supernatant, referred as Triton X‐100 soluble fraction (TSF), was stored at −20°C; the pellet, referred as PSD or Triton X‐100 insoluble fraction (TIF), was homogenized in a glass–glass potter in 20‐mM HEPES, protease, and phosphatase inhibitors and stored at −20°C in presence of glycerol 30%. Total proteins have been measured in the total homogenate and in the TIF fraction according to the Bradford Protein Assay procedure (Bio‐Rad, Milan, Italy), using bovine serum albumin as calibration standard.

Equal amounts of proteins of the homogenate (10 μg) and of TIF fraction (8 μg) were run on a sodium dodecyl sulfate‐8% polyacrylamide gel under reducing conditions and then electrophoretically transferred onto nitrocellulose membranes (GE Healthcare, Milan, Italy). Blots were blocked 1 h at room temperature with 10% nonfat dry milk in TBS +0.1% Tween 20 buffer and incubated with antibodies against the proteins of interest.

The conditions of the primary antibodies were the following: anti‐GLT1 (1:5,000, AbCam, UK), anti‐GluN1 (1:1,000, Invitrogen, Carlsbad, CA, USA), anti‐GluN2B (1:1,000, Santa Cruz Biotechonology, Santa Cruz, CA, USA), anti‐GluN2A (1:1,000, Invitrogen), anti‐SAP102 (1:1,000, Cell Signaling Technology Inc.), and anti‐β‐Actin (1:10,000, Sigma‐Aldrich).

Results were standardized using β‐actin as the control protein, which was detected by evaluating the band density at 43 kDa. Immunocomplexes were visualized by chemiluminescence using the ChemiDoc MP Imaging System (Bio‐Rad Laboratories). Gels were run two times each, and the results represent the average from two different runs.

### 2.5 Statistical analysis

Data were collected in individual animals (independent determinations) and are presented as means ± standard errors.

To enable visual comparisons across genotypes with different degrees of expression of glutamatergic synapse components, values are presented as percent of the control group, namely, the SERT^+/+^‐naive group that was not exposed to cocaine self‐administration. Molecular changes produced by genotype and cocaine exposure alone as well as by their combination were analyzed using a two‐way analysis of variance (ANOVA), with genotype and cocaine self‐administration as independent variables. When dictated by relevant interaction terms, Fisher's least significant difference (LSD) test was used to characterize differences among individual groups of rats. However, when no interaction between genotype and cocaine self‐administration was observed, only the main effects were reported. Significance for all tests was assumed at *p* < 0.05.

## 3 RESULTS

### 3.1 Cocaine intake

As we reported previously,^25^ no genotype differences were observed during the acquisition of cocaine self‐administration. Under ShA conditions, the daily number of cocaine infusion was higher in SERT^−/−^ versus SERT^+/+^ rats, leading to a higher total cocaine intake in SERT^−/−^ (172 ± 24 infusions) versus SERT^+/+^ (81 ± 13 infusions) rats. When the rats were allowed to self‐administer cocaine under LgA, the daily number of cocaine infusions was higher in SERT^−/−^ versus SERT^+/+^ rats, leading to a higher total cocaine intake in SERT^−/−^ (1,209 ± 88 infusions) versus SERT^+/+^ (823 ± 157 infusions) rats. Under both ShA and LgA conditions, the number of inactive lever presses were similar in both genotypes.

### 3.2 Expression levels of the glial glutamate transporter in the homogenate of ILc and PLc and following ShA and LgA to cocaine in SERT^+/+^ and SERT^−/−^ rats

**FIGURE 2:**
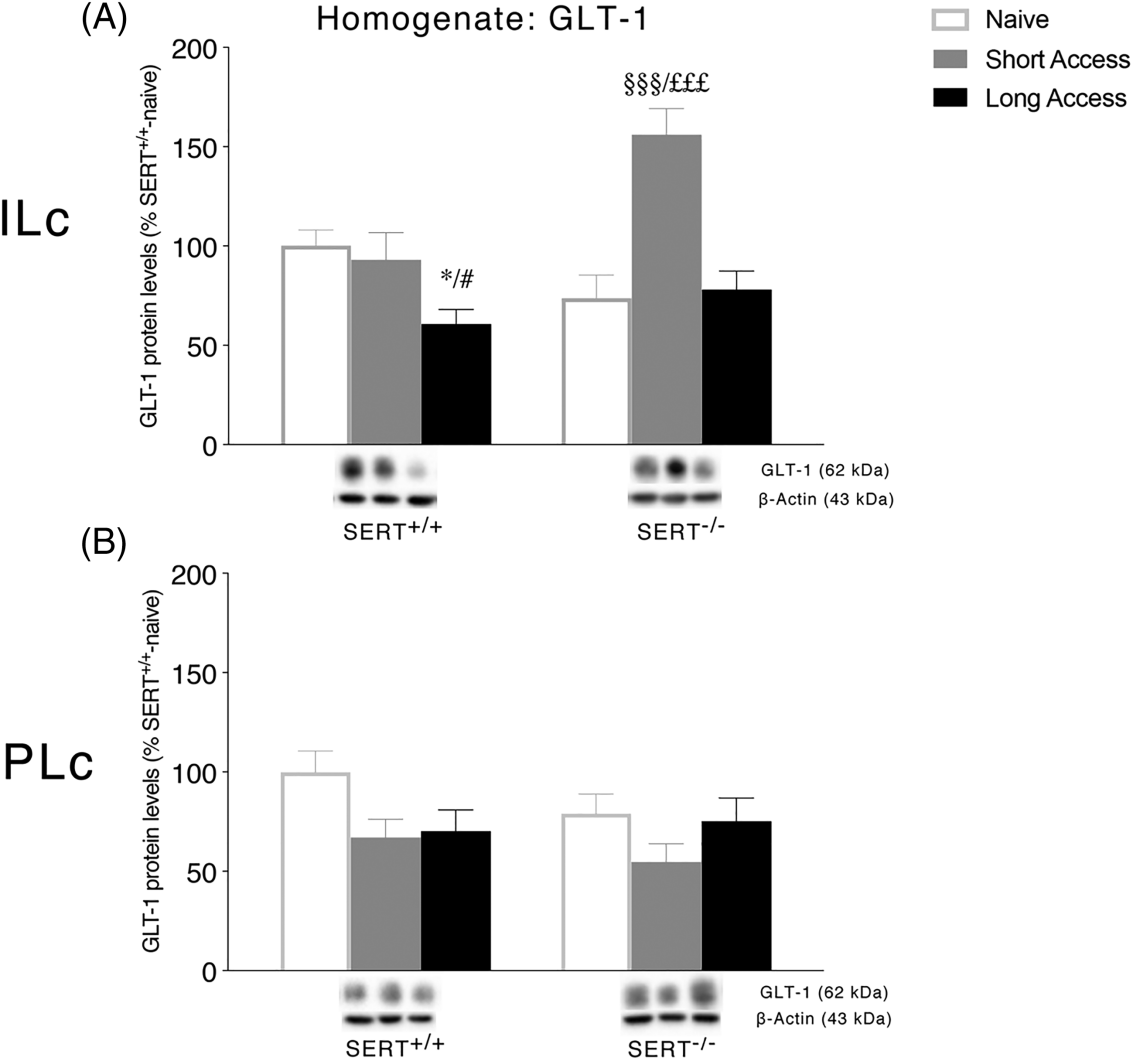
Interaction between serotonin transporter (SERT) deletion and cocaine self‐administration on the glial glutamate transporter 1 (GLT‐1) in the infralimbic (ILc) and prelimbic (PLc) cortices. Protein levels of GLT‐1 in ILc (Panel A) and PLc (Panel B) are expressed as percentages of SERT^+/+^‐naive rats. Below the graphs, representative immunoblots are shown for GLT‐1 (62 kDa) protein in the homogenate of ILc (Panel A) and PLc (Panel B), respectively. Histograms represent the mean ± SEM of five to six rats per group. ^*^ *p* < 0.05 versus SERT^+/+^ naive; ^#^ *p* < 0.05 versus SERT^+/+^ ShA; ^§§§^ *p* < 0.001 versus SERT^−/−^ naïve; ^£££^ *p* < 0.001 versus SERT^−/−^ LgA (two‐way ANOVA followed by Fisher's least significant difference [LSD] multiple comparisons test)

We first evaluated the expression of the main glial glutamate transporter responsible for the clearance of glutamate from the synaptic cleft, that is, GLT‐1, in the homogenate of ILc and PLc of SERT^−/−^ and SERT^+/+^ under naive conditions and following the different paradigms of cocaine self‐administration. In the ILc, two‐way ANOVA revealed a main effect of cocaine access (*F*_2,29_ = 13.24, *p* < 0.0001) and a cocaine access × genotype interaction (*F*_2,29_ = 8.666, *p* = 0.0011; Figure 2A). Further intergroup subtesting indicated that the ShA procedure significantly enhanced GLT‐1 expression in SERT^−/−^ rats (+82%, *p* < 0.0001 vs. SERT^−/−^ naive; +78%, *p* < 0.0001 vs. SERT^−/−^ LgA) but not in SERT^+/+^ rats (−7%, *p* = 0.6363 vs. SERT^+/+^ naive), whereas the LgA procedure significantly reduced GLT‐1 expression in SERT^+/+^ rats (−39%, *p* = 0.0143 vs. SERT^+/+^ naive; −32%, *p* = 0.001 vs. SERT^+/+^ ShA) but not in SERT^−/−^ rats (+4%, *p* = 0.681 vs. SERT^−/−^ naive). In the PLc, two‐way ANOVA of GLT‐1 revealed only a main effect of cocaine access (*F*_2,29_ = 3.787, *p* = 0.0346; Figure 2B). Due to its peculiar localization on astrocytes, no analyses were undertaken for GLT‐1 in the postsynaptic density.

### 3.3 Expression levels of GluN1 NMDA receptor subunits in the ILc and PLc homogenate and postsynaptic density following ShA and LgA to cocaine in SERT^+/+^ and SERT^−/−^ rats

**FIGURE 3:**
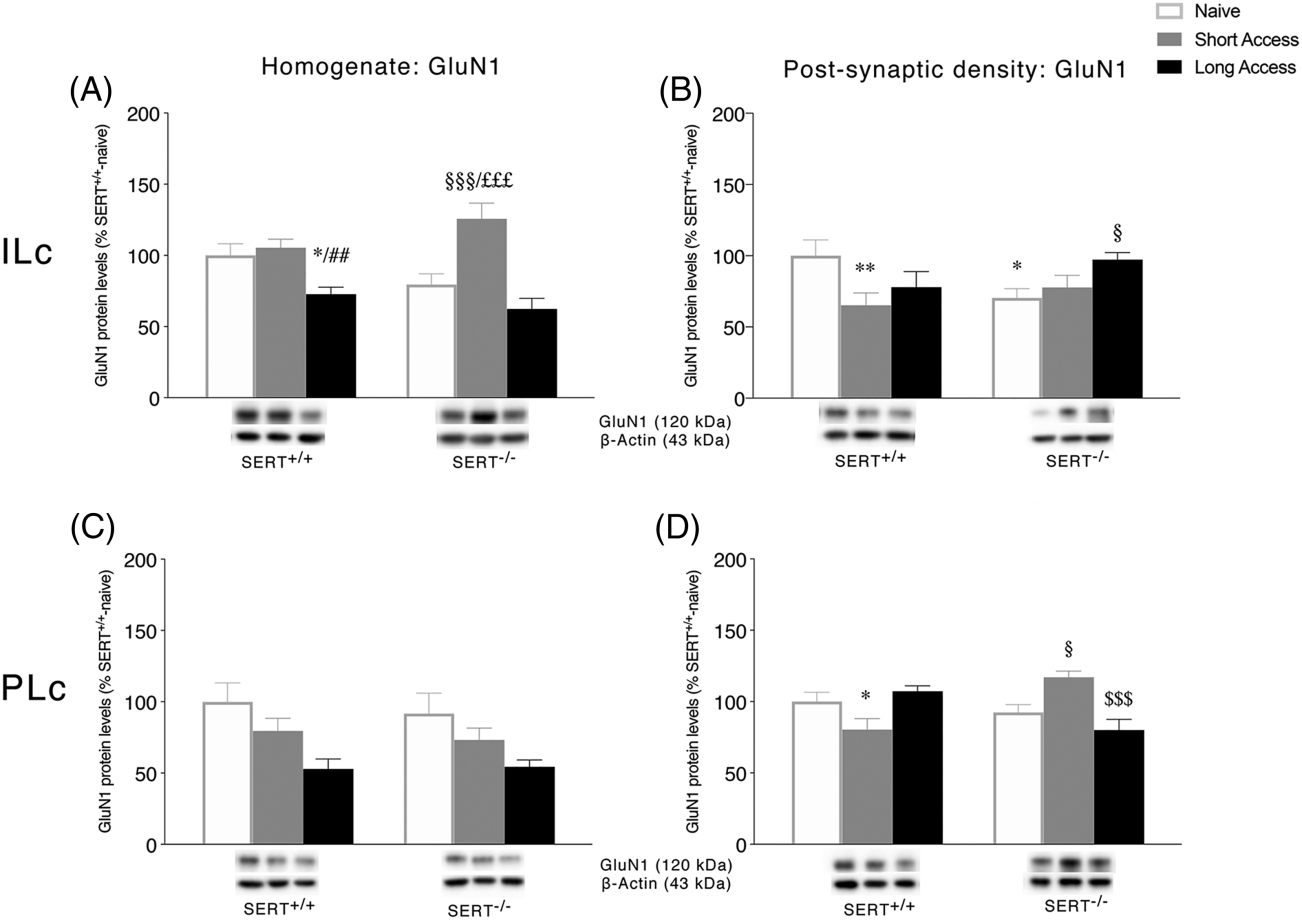
Interaction between serotonin transporter (SERT) deletion and cocaine self‐administration on the NMDA receptor obligatory subunit 1 GluN1 in the infralimbic (ILc) and prelimbic (PLc) cortices. Protein levels of GluN1 in the homogenate and postsynaptic density of ILc (Panel A,B) and PLc (Panel C,D) are expressed as percentages of SERT^+/+^‐naive rats. Below the graphs, representative immunoblots are shown for GluN1 (120 kDa) receptor in the homogenate and postsynaptic density of ILc (Panel A,B) and PLc (Panel C,D), respectively. Histograms represent the mean ± SEM of five to six rats per group. ^*^ *p* < 0.05, ^**^ *p* < 0.01 versus SERT^+/+^ naive; ^##^ *p* < 0.01 versus SERT^+/+^ ShA; ^§^ *p* < 0.05, ^§§§^ *p* < 0.001 versus SERT^−/−^ naïve; ^£££^ *p* < 0.001 versus SERT^−/−^ LgA (two‐way ANOVA followed by Fisher's least significant difference [LSD] multiple comparisons test)

We then analyzed protein expression of the obligatory subunit of the NMDA receptor, that is, GluN1, in the ILc and PLc of SERT^−/−^ and SERT^+/+^ under naive conditions and following different paradigms of cocaine self‐administration (Figure 3), in both the whole homogenate and the postsynaptic density. In the homogenate of the ILc, two‐way ANOVA revealed a main effect of cocaine access (*F*_2,29_ = 18.61, *p* < 0.0001) and a cocaine access × genotype interaction (*F*_2,29_ = 3.775, *p* = 0.0349; Figure 3A). Examining the individual treatment effects, similarly to GLT‐1, we found that the ShA procedure significantly enhanced GluN1 expression in SERT^−/−^ rats (+46%, *p* = 0.0002 vs. SERT^−/−^ naive; +64%, p < 0.0001 vs. SERT^−/−^ LgA) but not in SERT^+/+^ rats (+5%, *p* = 0.6256 vs. SERT^+/+^ naive), whereas the LgA procedure significantly reduced GluN1 expression in SERT^+/+^ rats (−27%, *p* = 0.0182 vs. SERT^+/+^ naive; −32%, *p* = 0.0055 vs. SERT^+/+^ ShA) but not in SERT^−/−^ rats (−18%, *p* = 0.139 vs. SERT^−/−^ naive). In the PSD of the ILc, two‐way ANOVA of GluN1 showed a significant cocaine access × genotype interaction (*F*_2,29_ = 4.443, *p* = 0.0207; Figure 3B). The deletion of SERT significantly reduced GluN1 localization in the PSD of naïve rats (−30%, *p* = 0.024 vs. SERT^+/+^ naive). Differently from the homogenate, the ShA procedure reduced GluN1 only in SERT^+/+^ animals (−35%, *p* = 0.0089 vs. SERT^+/+^ naive), whereas LgA increased the expression of GluN1 in SERT^−/−^ (+27%, *p* = 0.0492 vs. SERT^−/−^ naive) and not in SERT^+/+^ animals (−22%, *p* = 0.0855 vs. SERT^+/+^ naïve).

In the homogenate of the PLc, two‐way ANOVA revealed a main effect of cocaine access (*F*_2,29_ = 18.61, *p* < 0.0001) and of genotype (*F*_2,29_ = 18.61, *p* < 0.0001; Figure 3C), whereas in the PSD, two‐way ANOVA of GluN1 showed a significant cocaine access × genotype interaction (*F*_2,28_ = 12.0, *p* = 0.0002; Figure 3D). Interestingly, the ShA daily cocaine exposure differently influenced the expression of GluN1 in SERT^+/+^ versus SERT^−/−^ rats. In particular, the ShA procedure reduced GluN1 in SERT^+/+^ (−20%, *p* = 0.0323 vs. SERT^+/+^ naive) while increasing it in SERT^−/−^ animals (+25%, *p* = 0.0179 vs. SERT^−/−^ naïve; +37%, *p* = 0.0008 vs. SERT^−/−^ LgA). Moreover, LgA reduced GluN1 in SERT^−/−^ rats only (−37%, *p* = 0.0008 vs. SERT^−/−^ ShA).

### 3.4 Expression levels of GluN2A and GluN2B NMDA receptor subunits in the ILc and PLc homogenate and postsynaptic density following ShA and LgA to cocaine in SERT^+/+^ and SERT^−/−^ rats

**FIGURE 4:**
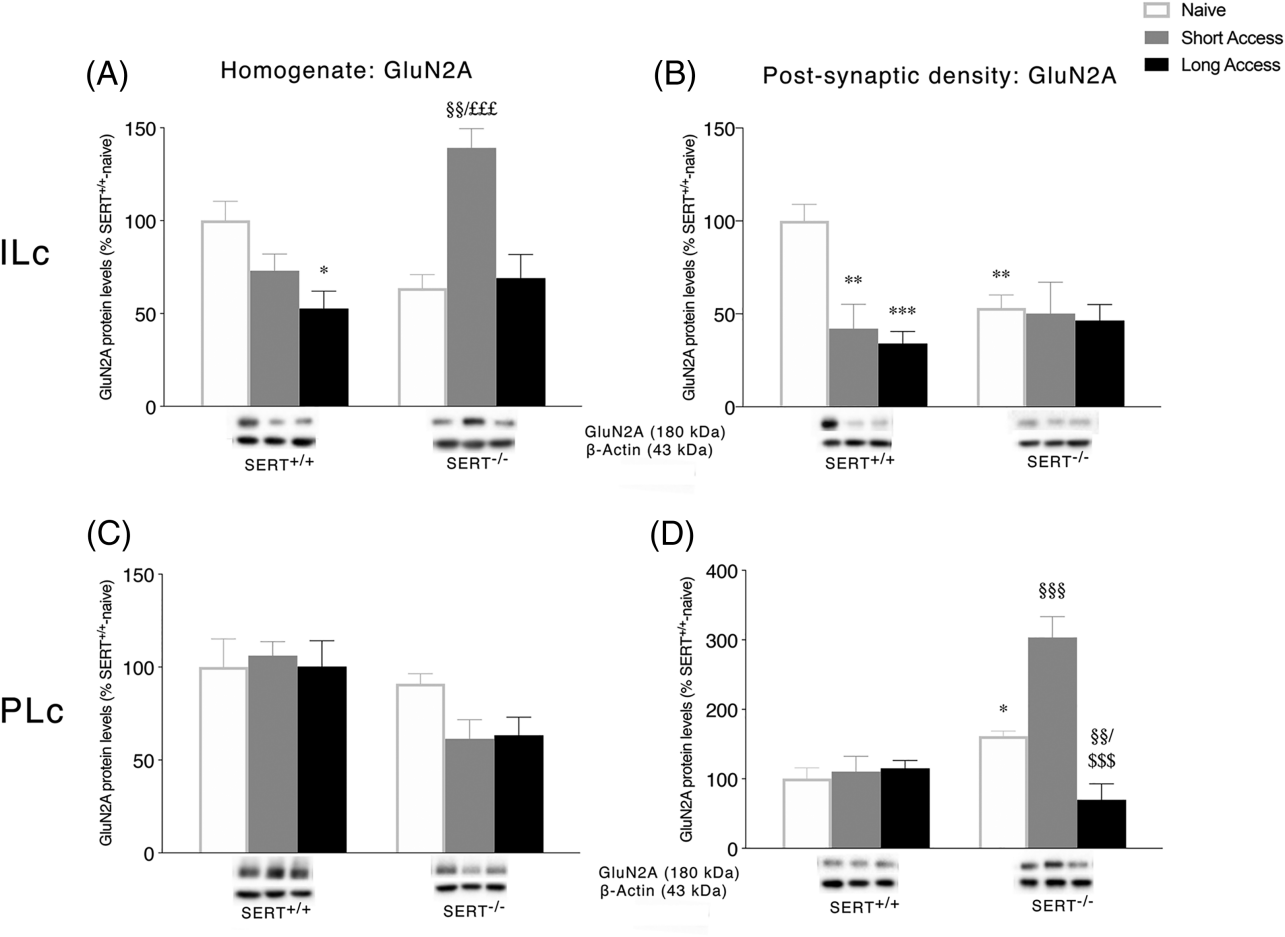
Interaction between serotonin transporter (SERT) deletion and cocaine self‐administration on the accessory NMDA receptor subunit 2A GluN2A in the infralimbic (ILc) and prelimbic (PLc) cortices. Protein levels of GluN2A in the homogenate and postsynaptic density of ILc (Panel A,B) and PLc (Panel C,D) are expressed as percentages of SERT^+/+^‐naive rats. Below the graphs, representative immunoblots are shown for GluN2A (180 kDa) receptor in the homogenate and postsynaptic density of ILc (Panel A,B) and PLc (Panel C,D), respectively. Histograms represent the mean ± SEM of five to six rats per group. ^*^ *p* < 0.05, ^**^ *p* < 0.01, ^***^ *p* < 0.001 versus SERT^+/+^ naive; ^§§^ *p* < 0.01, ^§§§^ *p* < 0.001 versus SERT^−/−^ naïve; ^£££^ *p* < 0.001 versus SERT^−/−^ LgA (two‐way ANOVA followed by Fisher's least significant difference [LSD] multiple comparisons test)

**FIGURE 5:**
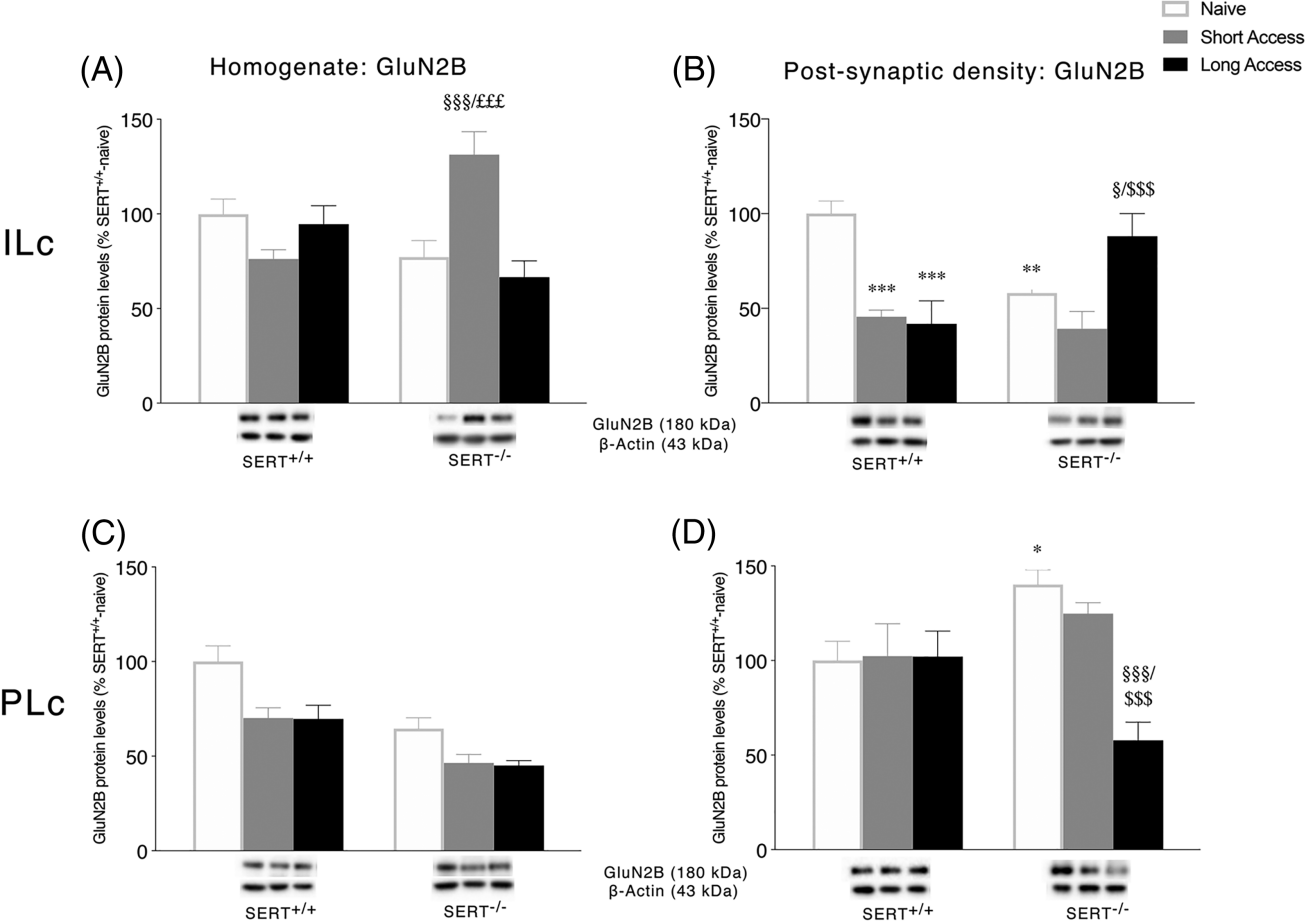
Interaction between serotonin transporter (SERT) deletion and cocaine self‐administration on the NMDA receptor subunit 2B (GluN2B) in the infralimbic (ILc) and prelimbic (PLc) cortices. Protein levels of GluN2B in the homogenate and postsynaptic density of ILc (Panel A,B) and PLc (Panel C, D) are expressed as percentages of SERT^+/+^‐naive rats. Below the graphs, representative immunoblots are shown for GluN2B (180 kDa) receptor in the homogenate and postsynaptic density of ILc (Panel A,B) and PLc (Panel C,D), respectively. Histograms represent the mean ± SEM of five to six rats per group. ^*^ *p* < 0.05, ^**^ *p* < 0.01 versus SERT^+/+^ naive; ^§^ *p* < 0.05, ^§§§^ *p* < 0.001 versus SERT^−/−^ naïve; ^$$$^ *p* < 0.001 versus SERT^−/−^ ShA; ^£££^ *p* < 0.001 versus SERT^−/−^ LgA (two‐way ANOVA followed by Fisher's least significant difference [LSD] multiple comparisons test)

Next, we investigated the expression of two accessory subunits of the NMDA receptor: GluN2A and GluN2B (Figures 4 and 5, respectively). Two‐way ANOVA of GluN2A and GluN2B expression in the homogenate of ILc revealed a main effect of cocaine access (GluN2A: *F*_2,28_ = 4.971, *p* = 0.0142; GluN2B: *F*_2,29_ = 3.368, *p* = 0.0484) and a significant cocaine access × genotype interaction (GluN2A: *F*_2,28_ = 6.648, *p* = 0.0043, Figure 4A; GluN2B: *F*_2,29_ = 13.79, *p* < 0.0001, Figure 5A). Further intergroup subtesting indicated that the ShA procedure significantly enhanced both GluN2A and GluN2B expression in SERT^−/−^ rats (GluN2A: +75%, *p* = 0.0011 vs. SERT^−/−^ naive; +70%, *p* = 0.0036 vs. SERT^−/−^ LgA; GluN2B: +54%, *p* = 0.0002 vs. SERT^−/−^ naive; +64%, *p* < 0.0001 vs. SERT^−/−^ LgA) but not in SERT^+/+^ rats (GluN2A: −27%, *p* = 0.6363 vs. SERT^+/+^ naïve; GluN2B: −24%, *p* = 0.0555 vs. SERT^+/+^ naive). Unlike the ShA procedure, the LgA procedure significantly reduced only GluN2A expression in SERT^+/+^ rats (GluN2A: −47%, *p* = 0.0108 vs. SERT^+/+^ naive; GluN2B: −6%, *p* = 0.6014 vs. SERT^+/+^ naive) but not in SERT^−/−^ rats (GluN2A: +5%, *p* = 0.7731 vs. SERT^+/+^ naive; GluN2B: −10%, *p* = 0.4398 vs. SERT^−/−^ naive). In the PSD fraction of the ILc, expression of GLuN2A and GluN2B revealed a significant cocaine access effect (GluN2A: *F*_2,28_ = 6.000, *p* = 0.0068; GluN2B: *F*_2,26_ = 9.407, *p* = 0.0008) and a cocaine access × genotype interaction (GluN2A: *F*_2,28_ = 4.318, *p* = 0.0232, Figure 4B; GluN2B: *F*_2,26_ = 12.93, *p* = 0.0001, Figure 5B). For GluN1 expression, the deletion of SERT significantly reduced GluN2A and GluN2B levels in naïve animals (GluN2A: −47%, *p* = 0.0068 vs. SERT^+/+^ naïve; GluN2B: −42%, *p* = 0.0017 vs. SERT^+/+^ naïve). In SERT^+/+^ rats, cocaine self‐administration, independently from the duration of the daily psychostimulant exposure, reduced protein levels of both subunits (GluN2A: −58%, *p* = 0.0011 SERT^+/+^ ShA vs. SERT^+/+^ naïve, −66%, *p* = 0.0003 SERT^+/+^ LgA vs. SERT^+/+^ naïve; GluN2B: −54%, *p* = 0.0002 SERT^+/+^ ShA vs. SERT^+/+^ naïve, −58%, *p* < 0.0001 SERT^+/+^ LgA vs. SERT^+/+^ naïve), whereas the LgA procedure increased selectively GluN2B in SERT^−/−^ rats (+30%, *p* = 0.0197 vs. SERT^−/−^ naïve, +49%, *p* = 0.0004 vs. SERT^−/−^ ShA).

In the homogenate of the PLc, two‐way ANOVA of GluN2A revealed only a genotype effect (*F*_2,28_ = 10.98, *p* = 0.0026, Figure 4C), whereas a significant genotype and cocaine access effect were observed for GluN2B expression (genotype: *F*_1,28_ = 31.71, *p* < 0.0001, treatment: *F*_2,28_ = 11.34, *p* = 0.0002, Figure 5C). In the PSD fraction of the PLc, two‐way ANOVA of GluN2A and GluN2B revealed a main effect of cocaine access (GluN2A: *F*_2,29_ = 18.21, *p* < 0.0001; GluN2B: *F*_2,28_ = 4.415, *p* = 0.0216), genotype (GluN2A: *F*_1,29_ = 19.98, *p* = 0.0001), and a genotype × cocaine access interaction (GluN2A: *F*_2,29_ = 19.18, *p* < 0.0001, Figure 4D; GluN2B: *F*_2,28_ = 4.868, *p* = 0.0153, Figure 5D). Interestingly, the deletion of SERT increased both GluN2A and GluN2B expression in naïve animals (GluN2A: +61%, *p* = 0.0286 vs. SERT^+/+^ naïve; GluN2B: +40%, *p* = 0.0308 vs. SERT^+/+^ naïve). Both ShA and LgA cocaine self‐administration did not alter GluN2A and GluN2B expression in SERT^+/+^ rats (GluN2A: +10%, *p* = 0.7003 SERT^+/+^ ShA vs. SERT^+/+^ naïve, +15%, *p* = 0.5796 SERT^+/+^ LgA vs. SERT^+/+^ naïve; GluN2B: +3%, *p* = 0.8887 SERT^+/+^ ShA vs. SERT^+/+^ naïve, +2%, *p* = 0.9020 SERT^+/+^ LgA vs. SERT^+/+^ naïve), whereas in SERT^−/−^ rats, the duration of the self‐administration sessions differently modulated NMDA subunits localization in the PSD. In these animals, GluN2A levels were increased following ShA (+143%, *p* < 0.0001 vs. SERT^−/−^ naïve, +234%, *p* < 0.0001 vs. SERT^−/−^ LgA) and reduced following LgA procedure (−91%, *p* = 0.0018 vs. SERT^−/−^ naïve). GluN2B levels, instead, were reduced only in SERT^−/−^ exposed to LgA sessions (−82%, *p* = 0.0004 vs. SERT^−/−^ naïve; −67%, *p* = 0.0134 vs. SERT^−/−^ ShA).

### 3.5 Expression levels of the scaffold protein SAP102 in the ILc and PLc homogenate and postsynaptic density following ShA and LgA to cocaine in SERT^+/+^ and SERT^−/−^ rats

To further characterize the impact of the combination of SERT deletion and cocaine self‐administration on the stability of NMDA receptor in the ILc and PLc, we evaluated SAP102 protein levels, a scaffolding protein that anchors and stabilizes NMDA receptors in the postsynaptic membrane.

**FIGURE 6:**
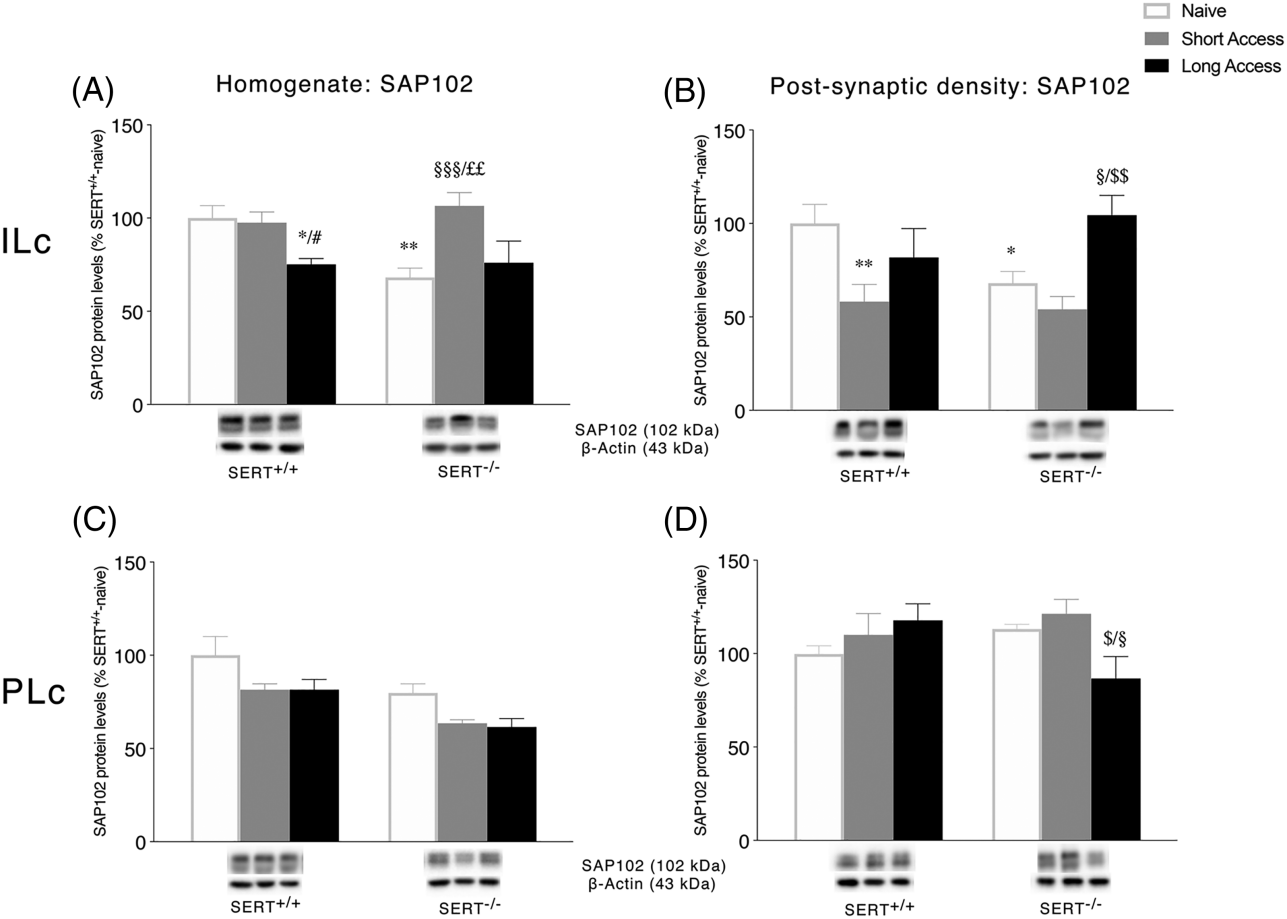
Interaction between serotonin transporter (SERT) deletion and cocaine self‐administration on the NMDA receptor‐related scaffolding protein SAP102 in the infralimbic (ILc) and prelimbic (PLc) cortices. Protein levels of SAP102 in the homogenate and postsynaptic density of ILc (Panel A,B) and PLc (Panel C,D) are expressed as percentages of SERT^+/+^‐naive rats. Below the graphs, representative immunoblots are shown for SAP102 (102 kDa) scaffolding protein in the homogenate and postsynaptic density of ILc (Panel A,B) and PLc (Panel C,D), respectively. Histograms represent the mean ± SEM of five to six rats per group. ^*^ *p* < 0.05, ^**^ *p* < 0.01 versus SERT^+/+^ naive; ^#^ *p* < 0.05 versus SERT^+/+^ ShA; ^§^ *p* < 0.05, ^§§§^ *p* < 0.001 versus SERT^−/−^ naive; ^$^ *p* < 0.05, ^$$^ *p* < 0.01 versus SERT^−/−^ ShA; ^££^ *p* < 0.01 versus SERT^−/−^ LgA (two‐way ANOVA followed by Fisher's least significant difference [LSD] multiple comparisons test)

In both homogenate and PSD fraction of the ILc, two‐way ANOVA of SAP102 revealed a main effect of cocaine access (homogenate: *F*_2,29_ = 8.072, *p* = 0.0016; PSD: *F*_2,28_ = 4.415, *p* = 0.0029) and a genotype × cocaine access interaction (homogenate: *F*_2,29_ = 7.256, *p* = 0.0117, Figure 6A; PSD: *F*_2,28_ = 3.419, *p* = 0.0469, Figure 6B). Further intergroup subtesting indicated that the deletion of SERT reduced both homogenate SAP102 expression (−32%, *p* = 0.0021 vs. SERT^+/+^ naïve) and its localization at the PSD (−32%, *p* = 0.0387 vs. SERT^+/+^ naïve). In line with the effects observed in NMDA receptor subunits levels, SAP102 expression is differently modulated by the duration of the self‐administration session. In particular, the ShA procedure was associated with an increase in SAP102 in the homogenate of SERT^−/−^ rats (+39%, *p* = 0.0003 vs. SERT^−/−^ naive; +31%, *p* = 0.0041 vs. SERT^−/−^ LgA), whereas the LgA procedure was associated with a decrease in SAP102 only in SERT^+/+^ rats (−25%, *p* = 0.0137 vs. SERT^+/+^ naive). In the PSD fraction, instead, exposure to ShA sessions reduced SAP102 levels in SERT^+/+^ (−42%, *p* = 0.0083 vs. SERT^+/+^ naive) but not in SERT^−/−^ rats (−14%, *p* = 0.3336 vs. SERT^−/−^ naive). The LgA procedure increased SAP102 expression only in SERT^−/−^ rats (+36%, *p* = 0.0199 vs. SERT^−/−^ naïve; +50%, *p* = 0.0022 vs. SERT^−/−^ ShA).

In the homogenate of the PLc, in line with observations for the NMDA receptor subunits expression, two‐way ANOVA of SAP102 showed a significant effect of cocaine access (*F*_2,28_ = 6.339, *p* = 0.0054) and genotype (*F*_1,28_ = 16.04, *p* = 0.0004, Figure 6C), whereas in the PSD fraction, a significant genotype × cocaine access interaction was observed (*F*_2,26_ = 4.471, *p* = 0.0214, Figure 6D). Examining individual cocaine access effects, SAP102 levels were reduced only in SERT^−/−^ rats following exposure to the LgA procedure (−26%, *p* = 0.0261 vs. SERT^−/−^ naïve; −35%, *p* = 0.0103 vs. SERT^−/−^ ShA).

## 4 DISCUSSION

Our data show that deletion of SERT influences the homeostasis of the glutamatergic synapse in both the ILc and PLc and confers a different reactivity to the two different cocaine exposure regimens. Taking together our behavioral and molecular findings, we depict a situation that points to the SERT as a master regulator of basal glutamate homeostasis, primarily in the ILc. Notably, its removal dictates a profile of responsivity to cocaine that is different based on the two subregions of the prefrontal cortex, the different modality of cocaine self‐administration as well as the cellular district taken into account.

In the ILc of SERT^+/+^ rats, we found that ShA cocaine did not affect the glial glutamate transporter GLT‐1, whereas LgA cocaine intake caused a decrease in GLT‐1 expression levels. Previous studies reported a reduced GLT‐1 expression in the nucleus accumbens after cocaine self‐administration for 2 h/day for 2 weeks combined with 3 weeks of extinction.^39^ Because this was associated with a decrease in glutamate uptake,^39^ it is possible that a decrease in GLT‐1 expression leads to an overflow of glutamate. Although cocaine exposure in a conditioned place preference test also decreased GLT‐1 in the nucleus accumbens, it did not affect GLT‐1 in the dorsomedial prefrontal cortex.^40^ Here, we extend the nucleus accumbens findings to the ILc.^41^

In the ILc of SERT^−/−^ rats, GLT‐1 protein levels were unaffected under baseline conditions, increased after ShA cocaine intake and normalized again under LgA conditions. The former may reflect an attempt to adjust homeostasis to the presence of cocaine, leading to a decrease in glutamate levels, whereas the latter may reflect a failure to maintain the adjustment. Intriguingly, in the ILc, hyperresponsivity of SERT^−/−^ rats to the ShA regimen is maintained in the glutamate markers here examined, suggesting an overall hyperactivation in the whole homogenate of SERT^−/−^ rats set in motion by ShA, that is, instead, lost under LgA. Based on previous studies, we hypothesize that the LgA cocaine intake in SERT^−/−^ rats may be driven by other factors (e.g., corticotrophin‐releasing factor [CRF] and brain‐derived neurotrophic factor [BDNF] changes in the central amygdala).^6^

Interestingly, this hyperactivation seen in the whole homogenate under ShA conditions disappears when examining the postsynaptic density. This is a critical point of discussion as the analysis of the homogenate and postsynaptic density provide different types of information on the homeostasis of the glutamate synapse: in fact, although changes in the whole homogenate reflect translational changes, in the postsynaptic density, they are indicative of altered localization. We hypothesize that the overall high peak in the expression of these glutamatergic molecular determinants following ShA may represent a (mal)adaptive response presumably secondary to decreased glutamate levels (see above), which may lead to increased synthesis of these glutamatergic proteins extrasynaptically. This may serve to regulate volume glutamate transmission but, perhaps, not translate to altered synaptic glutamate transmission. Thus, although the expression of NMDA receptor subunits is increased in the ILc postsynaptic density, such upregulation is not paralleled by an increased expression of the scaffolding protein specific for the NMDA receptor complex, that is, SAP102. This presumably leads to unstable synapses. Taken together, these results do indicate that, in the ILc of SERT^−/−^ rats, there is an overall dysregulation of the glutamatergic synapse.

The PLc is characterized by a different situation. In SERT^+/+^ rats, no changes in any of the glutamatergic components (except for GluN1 in the postsynaptic density) were found. In the homogenate of naïve SERT^−/−^ rats, no changes in glutamatergic system determinants were observed as well. Yet in the postsynaptic density, we found an overall trend towards increased expression of glutamatergic markers following ShA, which was significant for GluN2A and GluN2B. Because GLT‐1 levels were not altered in this brain subregion, we cannot point to the receptor upregulation as a neuroadaptive response to glutamate efflux. Hence, a different mechanism may come into play to foster such an upregulation. At variance from the ILc, the regulation of the glutamatergic markers following ShA or LgA in the in the homogenate of PLc is not significantly different between genotypes, with the exception of the GluN2A subunit. Conversely, in the PLc postsynaptic density of SERT^−/−^ rats, we observed an increase in the expression of GluN1 and GluN2A following ShA and a steep decline in all receptors following LgA, suggesting a bidirectional homeostatic regulation in response to LgA. This indicates that, in this fraction, the glutamate complex NMDA receptor/SAP102 is oppositely regulated by ShA or LgA and suggests that, in this subregion, ShA‐exposed rats are able to mount a neuroadaptive response that wanes following LgA, both in term of synthesis or receptor localization. Focusing instead on LgA‐exposed SERT^−/−^ rats, a clear discrepancy catches the eye; in fact, an increased localization and stabilization of GluN2B‐containing NMDA receptors and their related scaffolding protein SAP‐102 were observed in the postsynaptic density of ILc in SERT^−/−^ rats, whereas we found an opposite effect in the postsynaptic density of PLc in SERT^−/−^: because it has been shown that selective inhibition of GluN2B‐containing NMDARs in PLc prevents the BDNF‐mediated inhibition of cocaine seeking,^42^ it is tempting to speculate that the herein observed reduced localization and stabilization of GluN2B/SAP‐102 complex induced by LgA in the postsynaptic density of PLc in SERT^−/−^ promotes the escalation of cocaine seeking.

In conclusion, our findings suggest that SERT influences the homeostasis of the glutamate synapse both under basal conditions and following repeated cocaine self‐administration. From our data, it appears that removal of SERT reorganizes and destabilizes the cortical glutamate synapse, presumably providing a ground for the higher intake of cocaine exhibited by SERT^−/−^ rats. Because SERT^−/−^ rats consume a higher amount of cocaine, our study does not inform whether it is removal of SERT, exposure to cocaine, or a combination of both that have generated the observed pattern of glutamatergic changes in the Ilc and PLc. In case of cocaine effects, cocaine‐exposure‐specific changes in 5‐HT receptor expression may very well have contributed to the observed ShA and LgA effects in the glutamate system.^43^ Future research should address in more detail the mechanisms leading to the glutamatergic changes. The ILc is generally considered to inhibit subcortical areas, and the PLc to stimulate subcortical areas.^44^ Previous studies showed that impaired fear extinction in SERT^−/−^ rodents is associated with reduced expression of the neuronal activity marker c‐Fos in the ILc but not PLc,^45^ increased theta synchronization between the ILc and lateral amygdala,^46^ and an increase in the apical dendritic branches of ILc pyramidal neurons.^47^ These findings do point out that deletion of SERT affects the structure and function of the ILc, potentially comparable to the finding of reduced connectivity between the prefrontal cortex and amygdala reported in humans carrying the short allelic variant of the serotonin transporter promoter polymorphism.^48^ Because SERT^−/−^ rats not only show impaired fear extinction, but also an impairment in the extinction of cocaine‐seeking behavior after ShA and LgA cocaine self‐administration,^4^, ^49^ functional changes in the ILc may contribute to their difficulties to refrain from cocaine intake and thereby their increased cocaine intake specifically under LgA conditions. Further changes in the PLc postsynaptic density, such as decreased GluN2B receptor expression, may aggravate this effect.

## CONFLICT OF INTEREST

The authors declare no conflict of interest in relation to the work herein described.

## AUTHOR CONTRIBUTIONS

MV and BvR performed the animal studies. LC, FM, and FT performed the molecular analyses. LC and MV did the statistical analyses. LC, FM, FT, and BvR managed the literature searches. LC, MV, JH, and FF designed the study and wrote the protocol and interpreted the data. LC, MV, JH, and FF wrote the manuscript. All authors contributed to and have approved the final manuscript.
